# Possibilities of establishing a smallholder pig identification and traceability system in Kenya

**DOI:** 10.1007/s11250-019-02077-9

**Published:** 2019-09-16

**Authors:** Florence Mutua, Johanna Lindahl, Delia Randolph

**Affiliations:** 1 International Livestock Research Institute, P. O. Box 30709, Nairobi, 00100 Kenya; 2 Zoonoses Science Centre, Uppsala University, P. O. Box 70790, SE-750 07 Uppsala, Sweden; 3 Department of Clinical Sciences, Swedish University of Agricultural Sciences, P. O. Box 70790, SE-750 07 Uppsala, Sweden

**Keywords:** Smallholder pig systems, Animal identification, Traceability, Disease surveillance, Food safety, Welfare

## Abstract

Consumers have a right to safer foods, and traceability is one approach to meeting their expectations. Kenya does not have an operational animal traceability system, and while a few initiatives have been piloted, these have only focused on the beef value chain. In this paper, we begin a discussion on traceability in the pig value chain, with an initial focus on smallholder systems of Western Kenya. First, a background to local pig production is given, and a description of animal identification and traceability options applicable to these systems is explained. Based on this, a “butcher-to-farm” traceability system, with health, production and food safety as objectives, is discussed. Requirements for establishing such a system (including actor incentives) are additionally discussed. The proposed approach can be piloted in the field and findings used to inform the design of a larger pilot and possibly pave way for implementation of a national traceability system, in line with the guidelines provided by the World Organization for Animal Health (OIE). Organized systems in the area (including commercial producer and trader groups) would offer a useful starting point.

## Introduction

The rising demand for animal-source foods can be attributed to factors such as urbanization, increased incomes and population growth. This, as observed by Delgado (2003), is an opportunity for farmers, particularly those in developing countries, to improve their incomes and better their livelihoods. Pigs can contribute in meeting this rising demand (Mekuriaw and Asmare 2014). They do not require much land, are easy to keep, can utilize by-products and food waste and are known to reproduce multiple times in a year. The pig sector is growing in spite of the many challenges (Beltran-Alcrudo et al. 2017). Pig production in Western Kenya is constrained by factors such as the inadequate feeding (Mutua et al. 2012; Carter et al. 2013), less established marketing strategies (Kagira et al. 2010; Levy et al. 2013), poor breeding (Mutua et al. 2011c) and diseases, particularly African swine fever (ASF) and cysticercosis due to *Taenia solium*, which present production (ASF) and public health effects (cysticercosis). Antibiotic resistance is also an emerging public health issue.

African swine fever is characterized by high mortality and severe economic losses. It is endemic in Kenya (Gallardo et al. 2011; Okoth et al. 2013). Pigs get infected either by direct contact with infected pigs or when they consume meat and products infected with the virus, or mechanically through contact with humans and vehicles. Control is complicated. There are rapid tests that allow for early detection (Steinaa et al. 2016); however, no treatment and no vaccine exist for the disease. Eradication is a challenge (Beltran-Alcrudo et al. 2017) as some of the suggested options (biosecurity, containment of pigs, disinfection, etc.) are not practical in many rural communities. “Stamping out”, which involves culling of infected animals and those that have been exposed, and safe disposal of their carcasses (Beltran-Alcrudo et al. 2017), is also impossible.

Cysticercosis due to *T. solium* is an important zoonotic disease in the area. Adult tapeworms reside in the intestine of humans resulting in taeniosis. Those infected can contaminate the environment with the parasite eggs, especially in areas where hygiene and sanitation are poor. Pigs, particularly the free-ranging ones, are infected (and develop cysticerci) when they eat human faeces or feed contaminated with *T. solium* eggs. Infection in humans is through consumption of incompletely cooked pork (with cysticerci) which leads to tapeworm infection. Meat inspection procedures are expected to identify positive carcasses, but their low sensitivity is a challenge (Dorny et al. 2004). The risk of consuming infected pork is high; in Western Kenya, according to Thomas et al. (2017), any pork that is consumed locally has a 0.006 probability of containing at least one viable *T. solium* cysticercus. Humans who accidentally ingest *T. solium* eggs (or eat food contaminated with eggs from a person carrying a tapeworm) can develop cysticercosis and neurocysticercosis, an important cause of epilepsy in the developing world. Epilepsy in rural communities is difficult to diagnose and treat, often stigmatizes those affected, causes suffering and increases the cost of medication.

Investing in traceability is one option of addressing some of problems associated with the value chain (disease control, production, markets, pork safety, public health). The fact that farmers keep a few pigs at a time and rely on a marketing chain that is short and less complex makes this value chain suitable for implementation of the system. Once developed, the system can progressively be improved to address any emerging issues (production, health). Traceability relies on unique identification of animals, registration of actors, recording and establishment of a database to store the required information. It is important for surveillance, food safety and trade. With good records, farmers are kept aware of the sources of their pigs, the management activities (e.g. treatments) associated with each pig and whom the pig is sold to. Similarly, records can help determine sources of pigs being ferried by traders, those at the slaughterhouses/slabs, as well as the sources of carcasses found at butcher shops. In case of disease outbreaks, for example African swine fever, an operational traceability system could help identify problem farms and allow for measures to be put in place early enough to avoid further spread of the virus (thus reducing the loss that would otherwise be incurred if no measures are put in place to contain the disease). The system could also enable withdrawal of unsafe pork from the market (infected or contaminated carcasses) and add to initiatives that safeguard human health. Traceability systems can support trade at local, regional and international levels and are an incentive for farmers to explore and enter new markets.

Many developing countries still lag behind in the implementation of traceability systems (despite its growing global interest). In Kenya, a few systems have been piloted, but these have only focused on the beef value chain, and mostly in pastoralist areas. Investments on the pig sector, particularly those that are geared towards increasing production and improving safety, are promising. In this paper, we discuss traceability in the context of smallholder pig systems and use this as a basis to describe a system that is appropriate for the value chain in Western Kenya and which can be piloted in the field to assess its practical applicability. The participation of traders and other players in the value chain is key in the realization of such a system. Organized systems in the area including the more commercial smallholder farms and trader groups would be a useful starting point.

## Study approach

We conducted a desk review of literature on pork traceability as well as on pig production in Kenya. The results, together with authors’ experience on the topics, were used to inform the design of a traceability system for the smallholder value chain. An illustration of how the tool can be used to support surveillance of porcine cysticercosis and African swine fever is given.

## Results

### Description of the smallholder pig system in Kenya

Three pig production systems have been described (FAO 2012): intensive large-scale commercial farms, small-scale commercial farms and the traditional free-range system. Large pig farms are concentrated in Nairobi, Kiambu and Rift valley, and each owns between 5000 and 30,000 pigs.

Small-scale commercial farms keep a variable number of pigs, from less than 10 to 100 pigs. In most cases, farmers keep one sow and often adopt farrow-to-finish production approaches (Wabacha et al. 2004a); however, other arrangements such as the weaner-to-finish and farrow-to-weaner also exist (Mbuthia et al. 2015). The pigs are housed throughout the year and are stall fed 1–2 times a day (Mbuthia et al. 2015). Farm sizes are small, mixed farming is common, and farmers rely on family labour to manage the animals (Kirima et al. 2017). Farmers either buy complete feeds from millers and local retailers or buy the raw materials and use them to prepare feed rations on-farm (Mutua et al. 2012; Mbuthia et al. 2015). Supplementation with waste from market and boiled blood from slaughterhouses may also occur (FAO 2012). The main breeds are the large white and landrace (including their crosses) (Wabacha et al. 2004a; Kirima et al. 2017). A combination of different breed types was reported by Mujibi et al. (2018). Pigs are slaughtered and consumed either locally or sold in urban centres (FAO 2012; Kirima et al. 2017).

The traditional system is popular in Western Kenya (Mutua et al. 2011a, 2012; Mbuthia et al. 2015; Nantima et al. 2015a). Pigs are left to roam freely or are confined (either in stalls or by tethering). There is very little cash investment, herd sizes are small and women are involved in management (Mutua et al. 2011a; Nantima et al. 2015a). Sow owners will have their pigs bred by their neighbour’s boars and piglets sold within the village, typically at the age of about 2 months (Mutua et al. 2011c, 2012). Free-range pigs spend more time outside their homesteads of origin and have been shown to move an average of 4340 m in a 12-h period (Thomas et al. 2013). The pigs are maintained on local feed supplies (although a few farmers may supplement the sows and piglets) (Mbuthia et al. 2015). Swill feeding is common in urban and peri-urban areas (Murungi et al. 2015; Nantima et al. 2015b); swill ought to be treated to kill any pathogens that may be present (Muthuramalingam et al. 2011). Pigs may also scavenge on garbage pits (FAO 2012). They are a concern when they destroy crops and are sometimes confiscated and retained by the owners of the destroyed fields (Mutua et al. 2011a; FAO 2012). A few small-scale commercial farms exist in the region. Performance of traditionally reared pigs (in terms of weight gain) is often lower than that in the commercial systems (Wabacha et al. 2004b; Mutua et al. 2012).

Animal health services are mostly provided by private veterinarians (Wabacha et al. 2004a; Mbuthia et al. 2015), but farmers also have direct access to antibiotics (Muthuma et al. 2016). There is limited data on what antibiotics are used (Wilson 2018) which is not surprising given the nature of the value chain. The use of penicillin, tetracyclines, erythromycin and sulphonamides by pig farmers was reported in the pilot by Irungu et al. (2015). Using antibiotics to manage infections and failure to observe drug withdrawal periods before slaughter can result in pork with drug residue limits above the recommended levels. Antibiotic use is an important driver of antimicrobial resistance (Magouras et al. 2017).

### Pig marketing and slaughter

**Fig. 1:**
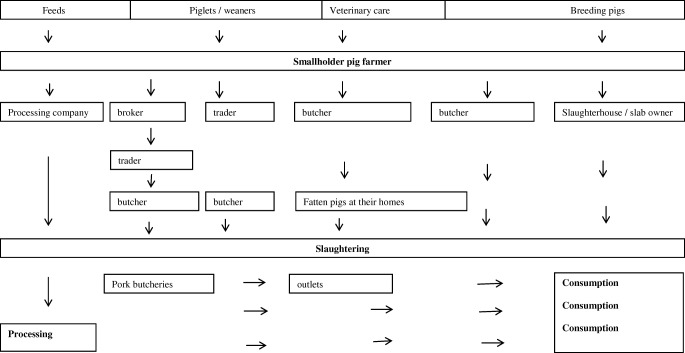
Overview of smallholder pig marketing in Western Kenya

The pig value chain in Western Kenya is summarized in Fig. 1. There are no physical markets for live pigs in the country, instead, buyers, mainly traders and middlemen, visit villages and farms sourcing for pigs to buy (Mutua et al. 2011b). Farmers may also contact traders when they have a need to sell a pig (Kagira et al. 2010). Pigs may be purchased daily (Levy et al. 2013) and, when bought, are transported from the farm direct  to the slaughter slab, either by trekking or using other available means (bicycles, motorcycles, etc.). Depending on the number bought, and season, buyers may choose to temporarily keep some of the pigs in their own farms awaiting slaughter. Middlemen (also known as brokers) buy pigs from the farms with the intention of selling them to butchers at a small fee (Kagira et al. 2010). Slaughter age is variable and depends on the needs of the farmer; weight assessments are done visually (Mutua et al. 2011b). The pork is sold raw, but some butcheries may also operate food outlets where cooked pork is sold (Levy et al. 2013). In the case of the larger (more commercial) farms, a major private sector firm, Farmer’s Choice Limited, enters into contractual arrangement with the farmers; the farmers are supported to raise the pigs, and the company commits to buying the pigs when they reach a certain market weight. Based on details available on the company website, there are nine pig purchase categories, which range from “class 1” which is a rate for pork to be exported, mainly within Africa and to Middle-East countries, to “class 9” which includes pork from culled sows. Pigs with dressed weight of ≤ 40 kg are not accepted by the processor. Sales through the processor gives smallholders an opportunity to have their pigs enter the export market chain.

Locally, marketing of pigs faces a number of challenges including conflicts with authorities because of non-compliance with regulations and poor transport infrastructure (Kagira et al. 2010). The Meat Control regulations (for local slaughterhouse, repealed in 2007) require food animals to be slaughtered in registered slaughterhouses, the carcass to be inspected by a qualified inspector and the meat to be identified by an official stamp that is specific for that slaughterhouse. Inspection procedures are as described in the Meat Control Act (Cap 356). Each slaughterhouse is expected to keep a daily recording of the number of animals slaughtered and condemnations done. Based on the Meat Control Regulations on transport of meat (1976), those transporting meat are required to apply for transport permits, which, when granted, are valid until the 31st December, the year of issue. The design of meat carriers should be as described in the regulation (of non-corrosive materials, easy to clean and disinfect, impermeable to water, and dust proof). Any meat on transport should be accompanied by a “certificate of transport”, a document that provides details of the meat being transported at any given time.

### Animal and public health issues

Biosecurity includes measures that restrict entry of disease agents into a farm, as well as those that prevent their spread to uninfected animals within the farm (FAO 2010). It is a challenge in many developing countries given the poor animal husbandry practices, absence of measures to restrict entry into the farms and the non-organized movement of animals (FAO 2012; Nabarro and Wannous 2014; Nantima et al. 2015b). In addition, smallholder systems offer little incentives for farmers to invest their time and resources on disease control (Beltran-Alcrudo et al. 2017), and recommended measures including segregation, cleaning and disinfection (FAO 2010) may not be feasible in a developing country context like Kenya.

African swine fever is endemic in many countries, and failure to confine pigs increases its risk of spread (Costard et al. 2009). The practice of buyers visiting several homesteads and villages in a day as they source for pigs (Mutua et al. 2011b) is a cause of concern. Moving pigs between herds for breeding can also contribute to ASF spread (Bellini et al. 2016). Farmers have a tendency to sell their pigs when an outbreak is suspected (Lichoti et al. 2017), a practice that would propagate the virus when affected animals get in contact with other pigs (FAO 2010; Beltran-Alcrudo et al. 2017). Further, meat from already infected pigs or those from pigs that die of the disease are a good source of the virus (Bellini et al. 2016). Thomas et al. (2016b) reported ASF virus  in healthy pigs at slaughter. Pig traders can minimize the risk of spreading ASF (and other diseases) by not accepting to buy sick animals and through observance of good biosecurity practices.

Cysticercosis due to *T. solium* has been reported in a number of studies, at prevalence rates of the following: 14% (*n* = 107, lingual test, Githigia et al. 2005), 9.8% (*n* = 316, lingual test, Mutua et al. 2007), 4% (*n* = 284, Ag Elisa, Kagira et al. 2010), 32% (*n* = 392, Ag Elisa, Eshitera et al. 2012), 37.6% (*n* = 343, Ag Elisa, Thomas et al. 2016a) and 4% (*n* = 700, Ag Elisa, Akoko et al. 2019). Some rural slaughterhouses are a risk to public health (Cook et al. 2017); hygiene is poor, ante-mortem inspections are lacking and slaughter of sick pigs is common. Some of the slaughter employees work while intoxicated (Cook 2014), and this likely increases their risk of exposure to important foodborne pathogens. Weak linkage between research, policy and extension, inadequate resource allocation and capacity building needs are additional issues (Maina 2015) requiring attention.

Pigs may also carry many other zoonotic diseases. Von Wissmann et al. (2011) reported *Trypanosoma brucei rhodesiense* in pigs in Western Kenya. Out of the 116 faecal and pig carcass swab samples analysed by Kikuvi et al. (2010), 13.8% were positive for salmonellosis. Haftman (2014) also analysed pig faecal samples for non-typhoid *Salmonella* species and reported a prevalence of about 20% (*n* = 195 samples in urban and peri-urban areas of Nairobi). Wilson (2018) reported non-typhoid *Salmonella* in 67% (*n* = 61) of pigs sampled in Busia (28% of the 39 positives were from faecal samples). Resistance of *Salmonella* isolates from pigs has been reported in few studies by Kikuvi et al. (2010) (for ampicillin, tetracycline and streptomycin) and by Haftman (2014) for resistance against sulfisoxazole (a sulphonamide) and ciprofloxacin. Multidrug resistance was recently reported by Wilson (2018).

Kenya has an Act (Cap 360) on animal welfare which prohibits handling of animals in a manner that causes them to suffer unnecessarily. Animal welfare data in the pig value chain in the country is however scanty. Welfare seems to be more of a concern in cases where pigs are not confined (as the pig is free and not under care of its owner). In some cases, farmers may use ropes to tie the pig for confinement, either on the legs or around the neck, but the tethers may not be properly made resulting to serious wounds (Mutua et al. 2011a).

### Animal identification methods

Old and new methods of animal identification co-exist in many countries (Caja et al. 2004). Hot-iron branding either on the skin, horns or hooves, with or without a written record of animal characteristics, is a very old tradition, initially applied on valuable animals (Blancou 2001). Tattooing, ear-notching, ear tagging and electronic identifiers are also frequently used (Caja et al. 2004). The World Organization for Animal Health (OIE) Terrestrial Animal Health code defines “animal identification” as the combination of identification and registration of an animal individually with a unique identifier or collectively by its epidemiological unit or group, with a unique group identifier, and “animal identification system” as the inclusion and linking of components such as identification of establishments, or owner, the person responsible for the animals, movements and other records with animal identification. Animal identification marks should be unique and visible. Automated techniques (including the use of machine-readable barcodes and radiofrequency identification (RFID)) allow for automation of traceability systems and promote faster and less expensive data collection processes (Stark et al. 1998). They allow for automatic reading of details, record large volumes of data and help reduce possibilities of errors during recording (Santamarina et al. 2007; Senk et al. 2013). RFID technologies use radio waves to automatically identify animals (McCathie 2004) and have the data stored in passive transponders. The system includes an identifier, a reader and a software to record and transfer data (Madec et al. 2001). Four types of RFID transponders are recognized (ICAR 2014): injectable transponders, electronic ear tags, bolus transponders (applicable only to ruminants) and instances where the transponder is used as an attachment to a visual ear tag. Ear tags come in different sizes, shapes and colours and can be plain or pre-numbered (Caja et al. 2004). Their certification considers factors such as the ease of application, efficiency, durability, how tamper-proof they are and effects on animal welfare and human health (ICAR 2018).

In pig systems, the use of ear tags, tattoos, ear notching and electronic devices is common in (Madec et al. 2001; Caja et al. 2004; Forsberg 2014). Tattoos are not appropriate in extensive systems as the numbers may become covered with soil making their readability difficult (Gosalvez et al. 2007). For injectable transponders, the site of device application should enhance readability, cause no harm to the animal and reduce loss of the device (Bortolotti et al. 2018). Skin characteristics may affect readability of transponders (Gosalvez et al. 2007). Good restraint and disinfection of sites are important in achieving successful application of transponders (Lamboolj et al. 1995). A transponder that is deposited close to the point of application is likely to be lost after application (Caja et al. 2005). Perineal transponders are difficult to recover in heavy pigs, and incisions to remove them at slaughter could compromise carcass quality (Prola et al. 2010). Intra-peritoneal application is perceived to be easy to apply (Marchi et al. 2007); Caja et al. (2005) found transponder application at the intra-peritoneal location (took 84.3 s) to take less time than that done at the auricle base which was more time consuming (101.7 s). On recovery, intra-peritoneal transponders are found loose in the peritoneal cavity and enveloped by abdominal viscera (Prola et al. 2010). There are safety concerns over the use of transponders in food animals (Caja et al. 2004; Gosalvez et al. 2007); however, Caja et al. (2005) and Santamarina et al. (2007) did not find any transponder in carcasses examined. In the study by Lamboolj et al. (1995), a small percentage of transponders injected at the base of the ears was unreadable at slaughter. The use of electronic ear tags is an option to avoiding transponder entry in food chain (Madec et al. 2001).

Electronic devices are expensive and likely out-of-reach of many smallholders. Less technical solutions may be more feasible for Kenya. Although ear tags are an option, their losses (or damage) during fattening, transportation and pig slaughter are a problem (Stark et al. 1998; Caja et al. 2005; Marchi et al. 2007). They are also likely to be tampered with given that they are visual. Their application is painful, and the large size, relative to that of the piglet’s ear, is also a concern (Hernandez-Jover et al. 2008; Leslie et al. 2010). In addition, records on ordinary ear tags can fade away with time and when used, their recovery may be compromised by pre-slaughter pig processes (Caja et al. 2005; Prola et al. 2010). Ear notching is affordable and easy to apply (Hernandez-Jover et al. 2008; Forsberg 2014). Using paint to temporarily identify animals is common, either for market animals (Mutua et al. 2018) or to enable differentiation of groups that have gone through certain management procedures (e.g. treated, vaccinated). Such marks should remain legible until the intended purpose has been achieved (Meisinger et al. 2008). Using skin and hair colour to identify pigs is not adequate for traceability (Madec et al. 2001; Gosalvez et al. 2007).

In Kenya, the Branding of Stock Act (Cap 357; revised in 2016) (GOK 2016) puts more emphasis on cattle identification; the second schedule of the Act states that “all cattle in the counties shall be identified by hot iron branding, coded ear tags, electronic transponders, either alone or in combination, or by any other method prescribed by the Director of Veterinary Services”. Directions for the brands and ear tag coding for all counties in the country are also given. In addition to the registered “brands”, the Act recognizes the use of “distinctive” marks on animals provided these do not resemble the already registered brands. Pigs reared in traditional systems are typically not identified, perhaps because they are kept in small numbers, which also makes it easy to identify and trace in case they get lost. Animal identification is also not well established even in the well established commercial systems; only a few (4%) of the farmers interviewed by Mbuthia et al. (2015) reportedly used ear tags (2% used ear notching). The use of ear notches in piglets was also mentioned in the FAO (2012) review. Pigs recruited for epidemiological research may be identified with ear tags to allow for follow up. Failure to identify animals at the farm level compromises disease control at the national level (Hernandez-Jover et al. 2008), and without proper recording, farmers may not know the sources of their pigs, whom the pigs were sold to, as well what management activities (e.g. treatments) have been given to each pig.

On its own, animal identification does not ensure traceability (Pavon 2011). The OIE’s Terrestrial Animal Health code defines traceability as “the ability to follow an animal or group of animals during all stages of its life” (OIE 2018a). Article 3 (15) of the EC regulations (No. 178/2002) defines traceability as “the ability to trace and follow a food, feed, food-producing animal, or substance intended to be, or expected to be incorporated into food or feed, throughout all stages of production, processing, and distribution”. Article 18 is specific on food and feed (EC 2002) and states that (1) “traceability of food, feed, food-producing animals, and any other substance intended to be, or expected to be incorporated into a food or feed shall be established at all stages of production, processing and distribution” and (2) “food and feed business operators shall be able to identify any person from whom they have been supplied with a food, a feed, or a food producing animal, or any substance intended to be, or expected to be incorporated into a food or feed”. With regard to imports, the regulation states that “food and feed imported in the community for placing on the market within the community shall comply with the relevant requirements of food law, or conditions recognized by the community to be at least equivalent thereto, or where a specific agreement exists between the community and the exporting country, with requirements contained therein”.

Traceability can be “forward” meaning that products are tracked through the entire food chain or “backward” which allows for products to be traced back to their sources (Senk et al. 2013). It is difficult to assure traceability post slaughter as methods used to identify animals may not be transferrable to the carcass (Mousavi et al. 2002; Forsberg 2014). Jensen and Hayes (2006) describe three systems of traceability: a more hypothetical one that relies on DNA sampling, a “farm to retail” system that preserves animal identity through slaughter to processing and a “batch” traceability system where the identity of the animal is maintained through to the carcass stage.

The demand for traceability is growing worldwide (Setboonsarng et al. 2009), and countries are at varying levels of its implementation, while some have mandatory regulations; others have voluntary systems that focus on export trade. Cattle identification is mandatory in EU, according to article 17 of the EC (2000) (no. 1760/ 2000) regulation “…. animals should be identified by an ear tag applied in each ear and in principle accompanied by a passport throughout any movement……”. The passport is issued for each animal to which an ear tag has been allocated and allows for recording of animal movements. Article 14 requires member states to create national computerized databases for recording of animal identity, holdings and movements of animals. The main reason for developing a traceability systems in the EU was to re-establish consumer confidence following the problem of Bovine Spongiform Encephalopathy (Pavon 2011).

Bowling et al. (2008) reviewed beef animal traceability in several countries. Botswana’s system was established in 2001 and relied on the use of rumen boluses. In addition to complying with export requirements, the system was also meant to address the problem of cattle theft. The device had details on both the owner (name, personal identification number) and the animal (sex, colour, location, etc.). Electronic ear tagging, with barcode and serial numbering, is the approved animal identification system in Namibia (Bowling et al. 2008). The tags are obtained either from the meat board or from the directorate of veterinary services. There is a primary electronic tag that is applied on the left ear and a secondary (visual) one which is applied on the right ear (MBN 2017). The Meat Board of Namibia manages the system (also known as the Farm Assured Namibian meat scheme). The database has brand, producer, traceability and import/ export information.

Meisinger et al. (2008) reviewed pig traceability in several countries. Australia, Brazil and Chile were found not to have mandatory systems. New Zealand had a mandatory “Animal Status Declaration” which captures a number of details (pig identification, owner, transport, treatments, movements, disease history). In Namibia, pigs are identified before 3 months of age and farmers are responsible for the identification (MBN 2017).

A few animal traceability interventions have been piloted in Kenya, but these have only focused on the beef value chain. One study, led by the International Livestock Research Institute, demonstrated the potential of using animal traceability systems to address surveillance and food safety needs in cattle traded in pastoralist areas (Mutua et al. 2018). Challenges associated with implementation of such a system in market animals (i.e. those related to bad weather, device application and data capture) were reported in the study. Traceability systems are also increasingly being applied in Kenya’s horticulture sector (Chemeltorit et al. 2018) with a focus on export trade.

### Design of smallholder pig traceability system

Methods used to identify pigs should allow for traceability in case of disease outbreaks and where food safety incidences are reported. Coded plastic ear tags may be the most suitable option considering their feasibility, costs and visibility. Activities need to be first captured in a “records book” issued to each actor and this is used to update the database if possible at the end of each day (at four levels—farm, trader, slaughter and butcher shops). The database design can follow the approach suggested by Mutua et al. (2018) but adapted to smallholder pig systems (with an initial focus on the organized systems). In the proposed approach, the research team designs the data capture and database system and, working with the local veterinary offices, provides the ear tags; farmers ensure the tags are applied to new pigs, while buyers, slaughterhouse operators and pork butcheries record and provide all the required documentation (Table 1). On arrival at the slaughterhouse, ear tag numbers are recorded and matched to respective carcasses and organs. Meat inspection reporting (as well as that for any sampling done) is also linked to the ear tag ID of the pig. As per the OIE’s Terrestrial Animal Health Code, design of animal traceability should specify the desired outcome, scope and performance. We consider animal health (to address ASF) and food safety (cysticercosis) as the main drivers for the system. Follow-up actions including institution of quarantine and stakeholder sensitization can be put in place once problem farms have been identified. For the scope, pigs kept on smallholder systems are the focus, and a butcher/retail to farm traceability is envisioned.

**Table 1:** Suggestions on what would be required to develop a suitable traceability system for the smallholder pig system in Kenya

Value chain actor	Description of the actor tasks	Incentive	Disincentive
Researchers	Designs and leads the implementation of pilot activities, in collaboration with local stakeholders	Donor funding	
Veterinary authorities at the county level (e.g. Kisumu)	Registers pig farmers, traders, butchers, slaughterhouse/slab owners	Better information on pig numbers. More information on disease	Additional work. No reward for having better information. Reporting diseases may cause problems
	Receives updates from the farmers and these are communicated to the project for updating of the online database system.		
Pig farmer	Identifies the pigs and keeps all the movement/activity records (sources of the pigs, any treatments given, details of breeding pigs, any deaths, pig sales including (dates, buyer).	May get support from veterinarians when they report	More effort
			More cost
		May get a higher price for an identified animal	May lack skills
			May get penalized if they report disease
	He/she notifies the local veterinarian/project whenever an event occurs.		May get penalized if it is discovered they sold a sick animal
Marketing/distribution	Identification of the buyer (broker, trader, etc.); date of the purchase; mode of transport including registration details of the motorcycle/vehicle used; details of where the pig is being taken next (a different farm for fattening, immediate re-sale, slaughter---gives slaughterhouse/ slab ID), any medication given.		More effort
			More cost
			May not want authorities to know about their activities
			May cause problems if they are identified to be transporting sick animals
Slaughter	Identification of the slaughterhouse/ slab; source—ID of seller; removal of the ear tags; meat inspection results (passed/not passed)/reasons; the pig ID number is issued and attached to records that accompany the carcass (the carrier and meat container details as listed in the “certificate of transport” document; retains the ear tag		More effort
			More cost
			May cause problems if they are identified to be slaughtering sick pigs
Butchery	Identification of the carcass (same as that for the live pig); if the carcass was shared among different butchers (ID of these butchers); date started selling the pork/end of sales for that carcass.	Can identify farms with problem pigs	More effort
		Can assure consumers the pigs are safe	More cost

Indicators such as the number of registered smallholder farms, number of pig’s ear tagged and activities captured in the system (sales, slaughter, death) can be defined, data collected and analysed to assess progress. An operational system is one that can trace cases (for example a carcass positive for *T. solium* cysts) back to their sources (e.g. farms).

### Case 1: use of traceability systems for surveillance and control of *T. solium* cysticercosis/taeniosis in communities

**Fig. 2:**
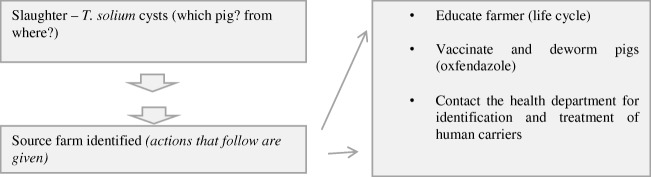
Application of traceability for surveillance of *T. solium* cysticercosis

We summarize this as Fig. 2. Positive pigs are identified at slaughter and the system is used to determine their source farms. Simple mitigation approaches such as raising awareness on the life cycle of the parasite can be implemented. Vaccination (to protect the pigs) and deworming (to treat sick pigs) approaches can be done where these are feasible. An optimal control programme should include detection and treatment of human tapeworm carriers (OIE 2018b); the medical department can be involved in the identification and management of the carriers.

Detection of cysts can be done by meat inspection, tongue palpation and diagnostic tests including ELISAs. The performance of these tests is variable; Dorny et al. (2004) reported 21%, 22%, 35% and 86% as sensitivities for the tongue, meat inspection, antibody ELISA and antigen ELISA respectively. The tongue test is perhaps the easiest to conduct in live pigs and can be used by farmers, traders and veterinarians to identify infected pigs (Guyatt and Fevre 2016). The ability to detect cysts during meat inspection largely depends on the nature of the incisions and infection load (easy in heavily infested pigs). OIE (2018b) (15.3.3) recommends disposal of entire carcass including viscera where *T. solium* cysts are found in multiple locations and inactivation where the cysts are localized, either by heat treatment to a core temperature of at least 60 °C or freezing to minus 10 °C or less for at least 10 days or any time and temperature equivalent.

### Case 2: use of traceability systems for surveillance and control of African swine fever in communities

**Fig. 3:**
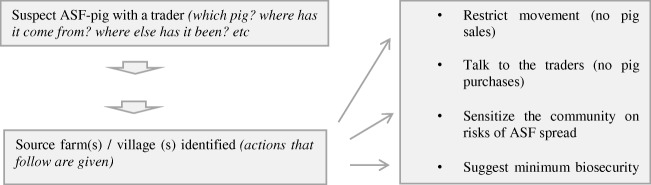
Application of traceability for surveillance of African swine fever

We summarize this as Fig. 3. Early detection of ASF improves the efficacy of the disease control measures (Bellini et al. 2016). Again, positive pigs are identified either during marketing or at slaughter and traceability is used to determine their source farms and villages. Government authorities are informed, and movement restriction is applied in the areas affected. Consequently, all stakeholders (farmers, traders, slaughterhouse personnel, etc.) are sensitized on measures to adopt to contain the problem. More epidemiological data is collected to characterize the virus.

## Discussion

Smallholder pig production is important for food security and income generation. Farmers are under pressure to satisfy the growing demand for food, and interventions to enable them achieve this are urgently needed. Food systems are also getting new players, making the chains long and complicated and increasing risks of important foodborne infections. As implied by Beltran-Alcrudo et al. (2017), it is very unlikely that the traditional ways of keeping pigs will change, and farmers may opt out of pig businesses if they are pushed to entirely confine pigs. Application of biosecurity measures in areas where pigs are not entirely confined is impossible (Bellini et al. 2016) and some diseases, particularly African swine fever, will continue to be a problem. The socio-economic impact of ASF outbreaks, at both farm and national level, is huge. Cysticercosis due to *T. solium* is an important zoonotic disease.

We have discussed traceability as an option to addressing many of the challenges associated with the pig value chain. There is no indication of the present development of such a system in the country. Animal identification is important for traceability, but based on our review, locally raised pigs are rarely identified. Once implemented, in addition to potentially increasing production (through reduced disease problems), the system could help ensure the quality and safety of pork sold in domestic market. Contaminated products can be tracked and removed from retail shelves, and problem herds can be traced and measures put in place to contain the problem. There is also an opportunity to open new market opportunities. Traceability relies on the use of unique numbers to identify animals, either individually, or as groups. In the smallholder context, individual identification is proposed given that farmers may source pigs from different farms and will only keep a few at a time (1–2 pigs) and sell them at different time periods. The “butcher-to-farm” traceability system we have described would allow for unsafe pork (e.g. that found with antibiotic residues beyond the recommended levels) to be traced to source farms and appropriate control measures be instituted. Similarly, at the slaughterhouse level, carcasses infested with *T. solium* cysts can be traced back to source farms. Indeed, raising community awareness and education on factors contributing to *T. solium* transmission are measures recommended by OIE’s Terrestrial Animal Health Code (OIE 2018b). Pigs found to manifest symptoms indicative of ASF, at any level in the marketing chain, can also be traced back to source farms (and villages), thus allowing for early containment of the virus spread and creating opportunities to train farmers about disease control and the role of biosecurity. Pigs found to have visible tether wound problems can be traced back to their previous owners who are then advised on how to better manage the pigs, including the welfare and food safety implications.

Since meat inspection in the country has now been taken up by the county governments, we see traceability as an option that counties, in partnership with the private sector, could use to market themselves as producers of “safe and traceable” pork. Farmers need to be sensitized on the importance of identifying animals and recording their movements and how this can improve market access. Beltran-Alcrudo et al. (2017) observed the need for trust by animal health officials and livestock traders, as it is for farmers. Such would encourage transparency along the market chain and ensure right data are collected and communicated. With funding support, this concept can be piloted in the field to assess its practical application and provide lessons to further enhance the tool. Smallholder farms with high likelihood of success (i.e. those with organized market chain) would provide a more appropriate starting point. The system can later be upgraded to include the use of automated technologies such as the electronic ear tags, but feasibility and cost factors will need to be considered. Implementation of traceability as a tool for health and food safety requires participation of all stakeholders in the value chain. Appropriate incentives would need to be explored to allow for widespread adoption of the intervention.
